# Clusters of postoperative endophthalmitis caused by non-tuberculous mycobacteria and *Pseudomonas aeruginosa*: diagnostic and sterilization challenges

**DOI:** 10.1186/s12348-026-00587-w

**Published:** 2026-06-13

**Authors:** Thibaud Mathis, Alper Bilgic, Lucas Sejournet, Perfecto Cagampang, Laurent Kodjikian, Aditya Sudhalkar

**Affiliations:** 1 Service d’Ophtalmologie, Hôpital de la Croix-Rousse, Hospices Civils de Lyon, 103, Grande Rue de la Croix-Rousse, Lyon, 69004 France; 2 Laboratoire MATEIS, UMR-CNRS 5510, INSA, Université Lyon 1, Villeurbanne, 69100 France; 3 OSG Augen-Tagesklinik Wittenberge, 19322 Wittenberge, Germany; 4 Manila Doctors’ Hospital, Manila, Philippines; 5 Retina Department, MS Sudhalkar Research Foundation, Baroda, 390001 India

## Abstract

**Purpose:**

To describe two consecutive postoperative endophthalmitis clusters—one caused by non-tuberculous mycobacteria (NTM) and the other by *Pseudomonas aeruginosa*—linked to a defective autoclave sterilization process in a single tertiary eye hospital in India.

**Methods:**

This retrospective study reviewed 25 cases of postoperative endophthalmitis between April and August 2024. The first cluster included 21 eyes infected with NTM, while the second involved 4 eyes infected with *P. aeruginosa*. Clinical data, microbiological findings, management strategies, and outcomes were analyzed. Environmental cultures and sterilization protocols were also reviewed to identify the source of contamination.

**Results:**

The first cluster presented insidiously, with symptoms appearing on average 40.4 ± 7.3 days postoperatively. Cultures revealed *Mycobacterium abscessus*, *M. fortuitum*, or *M. brisbaneii* in 18 of 21 eyes. All patients underwent pars plana vitrectomy with intravitreal vancomycin, ceftazidime, and dexamethasone, followed by oral linezolid. Visual recovery was favorable in most cases (20/21 eyes), with only one progressing to phthisis. The second cluster, occurring shortly thereafter, involved four cases of acute postoperative endophthalmitis caused by *P. aeruginosa*. Three eyes recovered useful vision, while one developed phthisis. Investigation revealed a defective autoclaving protocol as the common source of infection.

**Conclusions:**

These consecutive clusters highlight how minor deviations in sterilization protocols, compounded by high ambient humidity, can precipitate catastrophic outbreaks. Prompt identification, aggressive treatment, and reinforcement of sterilization standards prevented recurrence and resulted in excellent outcomes.

## Introduction

Postoperative endophthalmitis remains one of the most feared complications of intraocular surgery, often resulting in severe visual loss if not diagnosed and managed promptly [1]. While acute postoperative infections are typically easy to recognize, delayed-onset cases pose significant diagnostic challenges, particularly when caused by atypical organisms such as non-tuberculous mycobacteria (NTM) [2–4]. NTM species are free-living environmental organisms found in soil and water, distinct from the *Mycobacterium tuberculosis* complex and *M. leprae* [2]. More than 140 NTM species have been identified, and though pulmonary infections are most common, ocular involvement—especially postoperative endophthalmitis—has been increasingly reported [3–5]. These infections are often indolent, difficult to culture, and associated with poor visual prognosis if not treated early.

Autoclaving remains the cornerstone of surgical sterilization. When properly executed, steam sterilization ensures complete eradication of microorganisms, including spores [6]. However, even minor procedural lapses—such as incomplete closure of vent valves or insufficient drying—can compromise sterilization efficacy. In developing countries, where cost-saving re-sterilization of instruments is common, such oversights can have catastrophic consequences [7]. 

This study presents two consecutive clusters of postoperative endophthalmitis from a single charitable hospital in India: the first caused by NTM and the second by *Pseudomonas aeruginosa*. Both were traced to a defective autoclaving process. To our knowledge, this is among the largest reported series of NTM-related endophthalmitis and the only one to document consecutive outbreaks linked to a single source.

## Methods

This retrospective case series included all patients who developed postoperative endophthalmitis between April and August 2024 at a single center. The first cluster comprised 21 eyes infected with NTM; the second involved 4 eyes infected with *Pseudomonas aeruginosa*. All surgeries were performed by the same surgeon in the same operating room.

Data were extracted from medical records, including demographics, type of surgery, time between surgery and symptom onset, microbiological results, treatment, and visual outcomes. Environmental cultures were obtained from the operating theater, autoclave drums, sterilization area, and irrigation systems.

All patients underwent a complete ophthalmologic evaluation at presentation, including slit-lamp examination, intraocular pressure measurement, B-scan ultrasonography, and microbiological sampling of the vitreous. The primary outcome was final best-corrected visual acuity (BCVA). Secondary outcomes included microbiological identification, complications, and any required changes to sterilization protocols.

This study was conducted in accordance with the principles outlined in the Declaration of Helsinki. Informed consent was obtained from all patients included, and a local ethics committee (Ethics Committee for the Raghudeep and Sudhalkar Eye Hospitals, India) approved the study.

## Results

### First cluster: nontuberculous mycobacteria endophthalmitis

**Fig. 1:**
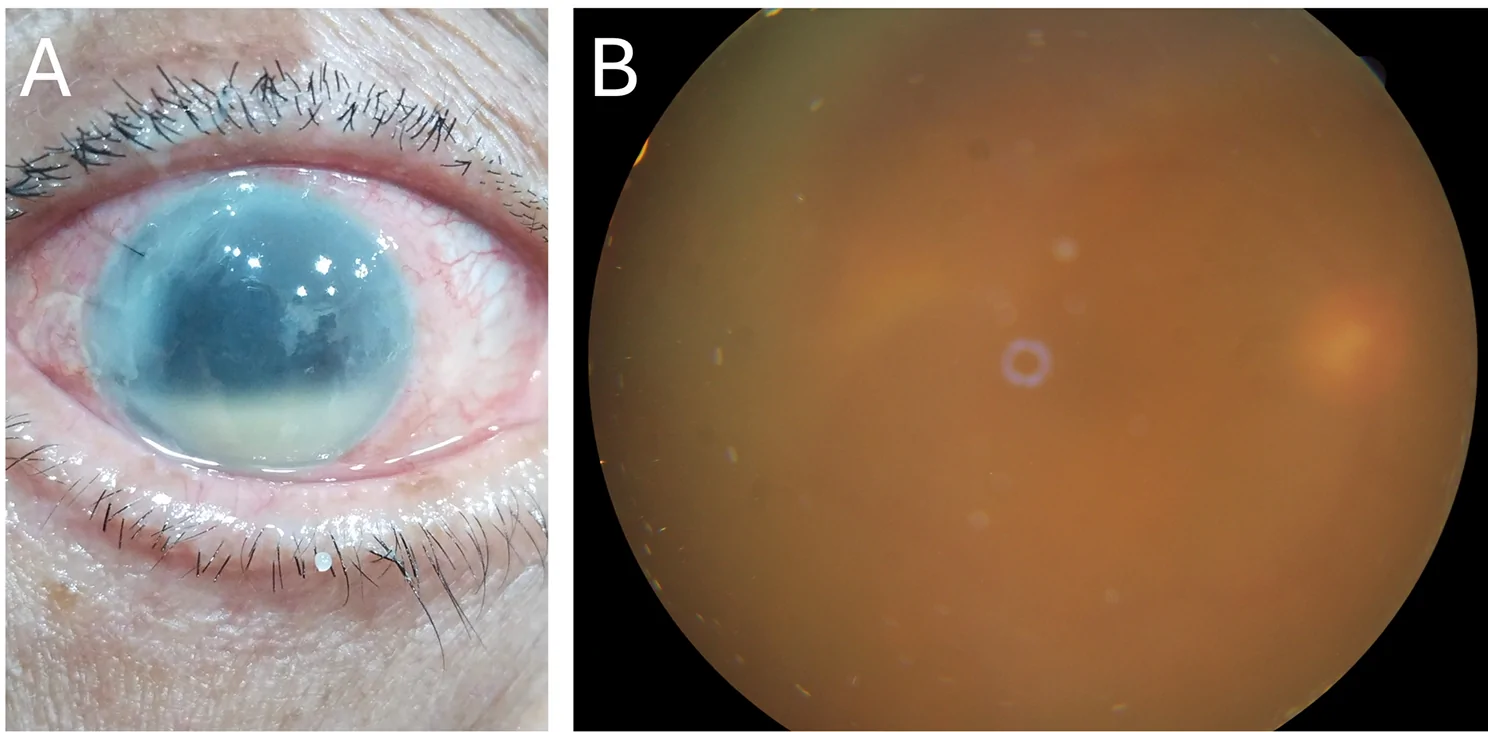
Clinical presentation of endophthalmitis. **A**. Corneal edema associated with hypopyon; **B**. Dense vitritis

Twenty-one eyes (21 patients) developed delayed-onset postoperative inflammation between April and June 2024. The mean age was 55.3 ± 4.2 years; 15 patients were male, and 9 (42.9%) had diabetes mellitus. Eighteen patients had undergone phacoemulsification with intraocular lens (IOL) implantation, while three had vitreoretinal surgery for retinal detachment. The mean interval from surgery to symptom onset was 40.4 ± 7.3 days (range 15–120 days). All patients reported decreased vision, and 80% also experienced ocular pain. On examination, anterior chamber reaction with hypopyon was present in all eyes, and 7 eyes had corneal edema. Fundus view was obscured in most cases due to dense vitritis (Fig. 1).

Vitreous samples were obtained during pars plana vitrectomy and cultured on blood and chocolate agar. Growth of NTM was confirmed in 18 eyes: *M. abscessus* (*n* = 8), *M. fortuitum* (*n* = 4), and *M. brisbaneii* (*n* = 6). Three eyes were culture-negative but showed identical clinical patterns (Table 1).

**Table 1:** Baseline characteristics of NTM cluster

Parameter	NTM cluster (*n* = 21)	*P. aeruginosa* cluster (*n* = 4)
Mean age, years (SD)	55.3 (4.2)	58.2 (3.8)
Sex ratio (M/F)	15/6	2/2
Diabetes mellitus, n (%)	9 (42.9%)	1 (25%)
Type of surgery, n (%)	Phaco + IOL: 18 (85.7%) VR surgery: 3 (14.3%)	Phaco + IOL: 2 (50%) VR surgery: 2 (50%)
Mean time to onset, days (SD)	40.4 (7.3)	1 day (1)
Culture positive cases, n (%)	18/21 (85.7%)	4 (100%)
Baseline BCVA, logMAR (SD)	0.64 (0.1)	0.56 (0.2)
Final BCVA, logMAR (SD)	0.3 (0.2)	1.0 (0.2)

BCVA: best-corrected visual acuity; NTM: non-tuberculous mycobacteria; Phaco + IOL: phacoemulsification and intraocular lens implantation; SD: standard deviation; VR: vitreoretinal

All patients received intravitreal vancomycin (1 mg/0.1 mL), ceftazidime (2.25 mg/0.1 mL), and dexamethasone (400 µg/0.1 mL) at the time of vitrectomy, followed by oral linezolid (600 mg twice daily for 7 days). Additional intravitreal injections (mean 1.6 ± 0.8) were given as needed. Four patients required IOL explantation to achieve complete resolution, and two underwent repeat vitrectomy. Corneal infiltrates occurred in four cases near the superior sideport; these were treated with intrastromal moxifloxacin.

At one month, BCVA improved to 20/30 in 7 eyes, 20/60 in 8 eyes, and 20/80–20/200 in 5 eyes. One patient progressed to phthisis bulbi. After one year, no recurrences were observed, and all but one eye maintained anatomical integrity (Table 2).

**Table 2:** Management and outcomes

Parameter	NTM cluster (*n* = 21)	*P. aeruginosa* cluster (*n* = 4)
Mean time to presentation	40.4 ± 7.3 days	≤ 2 days
Vitrectomy performed	21 (100%)	4 (100%)
IOL explantation	4 (19%)	0
Repeat surgery require	2 (9.5%)	1 (25%)
Phthisis bulbi	1 (4.8%)	1 (25%)
Final BCVA	20/30–20/200	20/63–HM

BCVA: best-corrected visual acuity; IOL: intraocular lens; NTM: non-tuberculous mycobacteria

### Second cluster: *Pseudomonas aeruginosa* endophthalmitis

In August 2024, four eyes operated within two consecutive days presented with acute postoperative endophthalmitis. Three patients presented within 24 h, while one, receiving oral steroids postoperatively, presented after five days with masked symptoms.

All patients underwent vitrectomy or vitreous lavage, and cultures confirmed *P. aeruginosa*, sensitive to ceftazidime and moxifloxacin. One patient required penetrating keratoplasty with silicone oil tamponade due to corneal infiltration and retinal detachment. Despite aggressive management, one eye progressed to phthisis, while the others recovered with final BCVA ranging from 20/63 to 20/80.

Investigation revealed that autoclave drums were being left vented after cycles, allowing moisture retention and bacterial survival. The combination of residual moisture and monsoon humidity likely promoted proliferation of both NTM and *Pseudomonas*. Following correction of the autoclaving protocol—closing vents immediately after cycles and allowing proper cooling—no further infections were reported over the subsequent 16 months.

## Discussion

This study describes an exceptional occurrence of two consecutive postoperative endophthalmitis clusters—one due to NTM and the other to *Pseudomonas aeruginosa*—originating from a single defective autoclaving process.

The delayed onset of the first cluster reflects the insidious nature of NTM infections, which often mimic low-grade postoperative inflammation. The organism’s ability to survive in moist environments explains its association with sterilization failures, particularly under humid tropical conditions [8]. Once identified, prompt vitrectomy combined with systemic and intravitreal therapy achieved remarkable outcomes, far better than those reported in most prior series, where final visual acuity was often ≤ 20/200 [3–5, 8–14].

The second cluster, caused by *P. aeruginosa*, presented acutely and was rapidly destructive, consistent with the pathogen’s known virulence [15]. In both outbreaks, early recognition and aggressive surgical debridement were critical in preserving anatomical integrity.

The investigation pinpointed a single error: autoclave vents left open after sterilization cycles, allowing condensation and bacterial survival. The issue was compounded by high ambient humidity during the monsoon season [8]. Once the procedure was corrected—closing vents immediately and implementing a 3-hour cooling interval—no further infections occurred.

Our findings reinforce the need for meticulous monitoring of sterilization protocols, particularly in high-volume centers. Regular staff training, humidity control, and microbiological surveillance are essential preventive measures.

Importantly, although sterilization procedures are typically delegated to operating room personnel and technicians, the ultimate responsibility for surgical safety remains shared with the operating surgeon. In high-volume surgical settings, even minor lapses in protocol adherence can have amplified consequences. Therefore, periodic training sessions and protocol audits—conducted at least annually or biannually—should be actively promoted and supervised by surgical teams to ensure strict compliance with sterilization standards. Such multidisciplinary oversight represents a critical safeguard against preventable outbreaks.

Although postoperative NTM endophthalmitis has been reported previously, this is, to our knowledge, the first documentation of consecutive NTM and *Pseudomonas* clusters linked to the same sterilization error. The outcomes observed here underscore the value of early, aggressive surgical intervention, guided antibiotic therapy, and institutional vigilance.

## Conclusion

Clusters of postoperative endophthalmitis due to NTM and *Pseudomonas aeruginosa* represent rare but catastrophic complications of ocular surgery. Even minor deviations in autoclave procedure can lead to widespread infection. Early recognition, prompt vitrectomy, and strict adherence to sterilization standards are essential to prevent similar outbreaks and safeguard patient vision.

## Data Availability

No datasets were generated or analysed during the current study.
